# Skyrmion electrical detection with the use of three-dimensional Topological Insulators/Ferromagnetic bilayers

**DOI:** 10.1038/s41598-017-17727-x

**Published:** 2017-12-19

**Authors:** Dimitrios Andrikopoulos, Bart Sorée

**Affiliations:** 1 KU Leuven, ESAT, Kasteelpark Arenberg 10, Leuven, B-3001 Belgium; 2 imec, Kapeldreef 75, 3001 Leuven, Belgium; 3 Universiteit Antwerpen, Physics Dpt., Condensed Matter Theory, Groenenborgerlaan 171, Antwerpen, B-2020 Belgium

## Abstract

The effect of the magnetic skyrmion texture on the electronic transport properties of the TI surface state coupled to a thin-film FM is numerically investigated. It is shown that both Bloch (vortex) and Néel (hedgehog) skyrmion textures induce additional scattering on top of a homogeneous background FM texture which can modify the conductance of the system. The change in conductance depends on several factors including the skyrmion size, the dimensions of the FM and the exchange interaction strength. For the Néel skyrmion, the result of the interaction strongly depends on the skyrmion number *N*_*sk*_ and the skyrmion helicity *h*. For both skyrmion types, significant change of the resistance can be achieved, which is in the order of kΩ.

## Introduction

Magnetic skyrmions^1–3^ are topologically protected whirling spin configurations that have been predicted since the end of the 1980s^4,5^. Their small size and robustness to defects makes them promising candidates for spintronics applications^6–9^. The main mechanism that gives rise to skyrmions is the competition between the Dzyaloshinskii-Moriya interactions (DMI) and exchange interactions. The direct experimental observation of skyrmions occured recently in chiral magnets and other B20-type materials where the DMI occurs due to the lack of inversion symmetry of the crystal^3^. Examples of such materials are MnSi^10^, Fe_1−*x*_Co_*x*_Si^11^, FeGe^12^ and Mn_1−*x*_Fe_*x*_Ge^13^. More interesting for technological applications however are ultrathin heavy metal/ferromagnetic films where the breaking of inversion symmetry at the interface and the large spin-orbit coupling from the heavy metal atoms lead to a sufficient DMI in order for Néel skyrmions to occur. Interfacial skyrmions have been demonstrated in epitaxial ultrathin films of Fe or PdFe monolayers on Ir (111)^14,15^, sputtered Pt/Co/MgO nanostructures^16^, Pt/Co/Ta and Pt/CoFeB/MgO nanostructures^17^ and Ir/Fe/Co/Pt nanostructures^18^. Room-temperature observation has also been made possible^16–19^, paving the way for the design and fabrication of skyrmion-based devices.

To this end, control of the skyrmion state is required. More specifically, skyrmion-based devices would require efficient skyrmion creation and annihilation as well as efficient read-out of the skyrmion presence or absence. The engineering of skyrmions has been studied by many authors^15,20–23^. Similarly, there have been proposals for electrical skyrmion detection using both in-plane and out-of-plane current^24–28^. While the out-of-plane electronic current techniques take advantage of the spin-mixing magnetoresistance^24,27,28^ to identify the skyrmion presence, the in-plane current techniques^25,26^ employ the emergent magnetic field of the skyrmion. This emergent field is attributed to the non-trivial real-space Berry curvature that the conduction electrons feel, leading to the Topological Hall Effect (THE)^29^.

In this work, we combine the non-trivial skyrmion magnetization with a material that has brought a lot of attention in the spintronics community, namely topological insulators (TI)^30–32^. These are materials which insulate in the bulk, but provide conducting edge (2D TIs) and surface (3D TIs) modes, which are spin-polarized. Electrons populating those states have their momenta and spins locked perpendicularly to each other. Consequently, processes that do not affect the electron spin cannot have a major impact on the momentum, strongly suppressing backscattering in this way. Our motivation of combining skyrmions with the surface states of TIs stems from the magnetization texture of the skyrmion itself: the magnetization texture in proximity leads to spin-exchange interactions with the surface state electrons, where the in-plane magnetization components, ${{\bf{m}}}_{\parallel }({\bf{r}})$ can be regarded as a local emergent magnetic induction field B^*e*^ interacting with the Dirac electrons^33^, while the out-of-plane magnetization component, *m*_*z*_(r), results in a real-space modulation of the mass term in the Dirac equation that describes effectively the surface states of a 3D TI.

**Figure 1:**
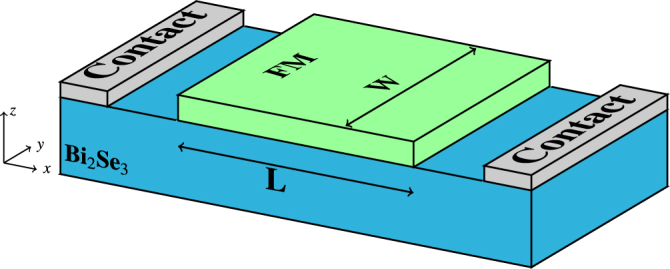
TI/FM bilayer of the present study. The FM layer is of length *L* nm and both the TI surface and FM are of width *W* nm.

**Figure 2:**
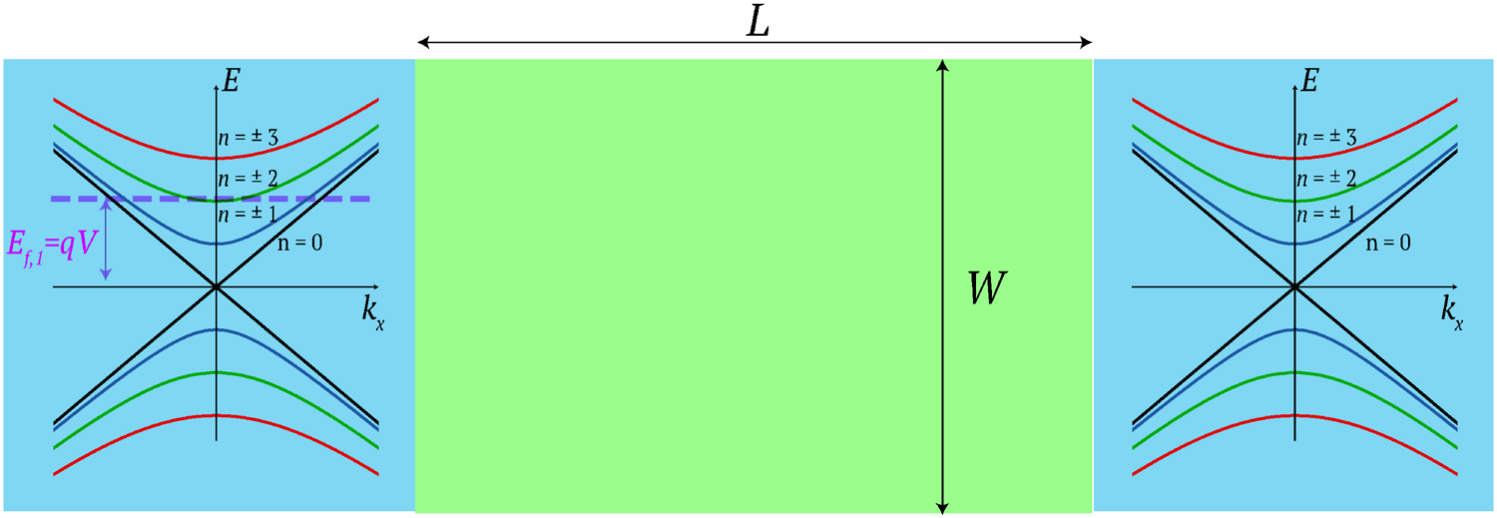
Top view of the system. In the “free” TI regions (blue color), the band diagram *E*_*n*_(*k*_*x*_) has been embedded. In the left contact which is considered as the input, a negative voltage *V* has been applied, such that the fermi level *E*_*f*,1_ = *qV* > 0. The fermi level at the right contact, *E*_*f*,2_ = 0 eV.

The aim is to numerically investigate the effect of the skyrmion texture on the electronic transport properties of the Dirac electrons. The proposed system setup is depicted in Figs 1 and 2. The system is composed of a TI/FM bilayer. For the present work, we have used as an example one of the most well-studied TIs, Bi_2_Se_3_^34–36^, while the FM can be any magnetic system supporting individual skyrmions (e.g. Fe, PdFe, Co^14–16,19^). We do not focus on how the skyrmion can be created in the first place, so for the present work we assume that there is also an interface with a heavy metal providing sufficiently strong DMI for skyrmion creation. Several methods for skyrmion nucleation have been proposed including spin current injection^37,38^, the use of an STM tip^14^, spin waves^39^, local heating via laser pulses^21^, domain-wall pairs^40^ as well as exploiting the device geometry^41^. In contrast to previous works^24–26,42^ we do not attribute the skyrmion presence to a Hall conductance signal, but rather study the longitudinal electronic transport properties of the TI surface state.

More specifically, we calculate the longitudinal conductance for the case where only a uniformly magnetized FM is present with magnetization texture ${\bf{m}}({\bf{r}})=\hat{{\bf{z}}}$ and compare it to the case when also a skyrmion is present. For simplicity we regard the skyrmion as a fixed texture, with its center coinciding with the center of the FM. We show that the skyrmion textures can lead to a change of the longitudinal resistance of the order of k Ω. This change is attributed to the additional scattering that the surface electrons feel from the change of the Dirac mass term and to the coupling of the in-plane magnetization components. Although the in-plane components do not modify the energy gap, their specific texture can significantly alter the system conductance due to the specific spin-momentum locking mechanism of the TI surface.

This work is organised as follows: in section 3 we give a detailed description of the proposed setup for skyrmion electrical detection. Then in section 4 we present the results of our simulations where we treat different parameters of our system setup including the dimensions of the system, the skyrmion size and type and the exchange interaction strength. Finally, in section 5 we highlight the most important results of this work and compare the detection mechanism with other recently proposed schemes.

### Proposed System Setup

The TI/FM bilayer is shown in Figs 1 and 2. The thin-film FM covers a rectangular region of length *L* and width *W* of the TI surface. For a free TI surface, the effective surface state Hamiltonian is $H={v}_{F}({\bf{p}}\times {\boldsymbol{\sigma }})\cdot \hat{{\bf{z}}}$ with *v*_*F*_ being the Fermi velocity of the surface states and the eigenstates are spinor wavefunctions $${\rm{\Psi }}={(\begin{array}{cc}{\psi }_{a}(x,y) & {\psi }_{b}(x,y)\end{array})}^{T}$$. Under the influence of the FM, the magnetization texture couples to the spin of the electron on the TI surface via proximity-induced exchange interaction. Consequently, the effective surface state Hamiltonian for an electron on the TI surface is modified as follows^33,43^

1 $$H={v}_{F}{({\bf{p}}\times {\boldsymbol{\sigma }})}_{z}-{J}_{S}{\bf{m}}(x,y)\cdot {\boldsymbol{\sigma }}$$

where *J*_*S*_ is the exchange interaction strength and **m**(*x*,*y*) is the normalized, three-dimensional magnetization field vector of the FM. For the present study we use *v*_*F*_ ≈ 6 × 10^5^ m s^−1^ which corresponds to the Fermi velocity of one of the most well studied TIs, namely Bi_2_Se_3_^36^. In literature, values for the exchange interaction strength range from 5–50 mV^44–53^ and is determined by the interface of the TI/FM. In order to make our results as general as possible, we use in our simulations two values of the exchange interaction strength, namely *J*_*S*,1_ = 25 meV and *J*_*S*,2_ = 40 meV.

In Fig. 2, the top view of the TI surface and the energy dispersion of the free TI regions are illustrated. Due to the periodic boundary conditions along the transverse direction, which we use to emulate the effect of the conducting side-surfaces of the three-dimensional TI, sub-bands are formed resulting from the quantisation of the transverse momentum *k*_*y*,*n*_ = 2*nπ*/*W* with $n\in {\mathbb{Z}}$. For example, for *W* = 20 nm, the spacing between the energy sub-bands is of the order of 10^−1^ eV. The effective Hamiltonian (1) to derive the energy dispersion, is just an approximation which is valid in the vicinity of the Dirac point. In^34^ it is shown exprimentally that the linear dispersion is valid for energies *E* ≈ 300 meV from the Dirac point. A more accurate description of the surface state can be derived by incorporating hexagonal warping effects^35^. Due to the fact that the interaction strength is of the order of $25 \sim 40$ meV, we expect that the most effective interactions occur for energies $E \sim {J}_{S}$. For our transport problem, we concentrate on the low-energy regime and thus the approximation of using a Dirac cone dispersion (1) is valid.

## Results

### Reference texture

For our electrical skyrmion detector, we are going to use as reference conductance *G*_*R*_, the one corresponding to a trivial FM texture without any skyrmion present, where the magnetization is uniform and in the positive $\hat{{\bf{z}}}$ direction. In that case, ${\bf{m}}({\bf{r}})=\hat{{\bf{z}}}$ and the interaction term in Eq. (1) reduces to *J*_*S*_ *σ*_*z*_ allowing us to find analytical solutions (see section 6). This additional term opens a gap in the Dirac cone at the Γ point. Because this gap closes again in the free TI regions, which we regard as contact regions, the trivial texture is in essence a constant energy barrier for electrons. Consequently, for barriers extending further along the longitudinal direction and/or high barriers we expect a lower transmission probability for electrons with energies in the tunneling region, i.e. *E* < *J*_*S*_. This transmission probability in turn, affects the conductance of the system. This is clearly seen when only one transverse mode is injected at the input because in this case, the conductance of the system and the transmission probability coincide in the low-temperature regime with *G* = d*I*/d*V* = *q*/*h T*_0_(*E*_*f*_) (see section 6 for details).

### Skyrmion textures

**Figure 3:**
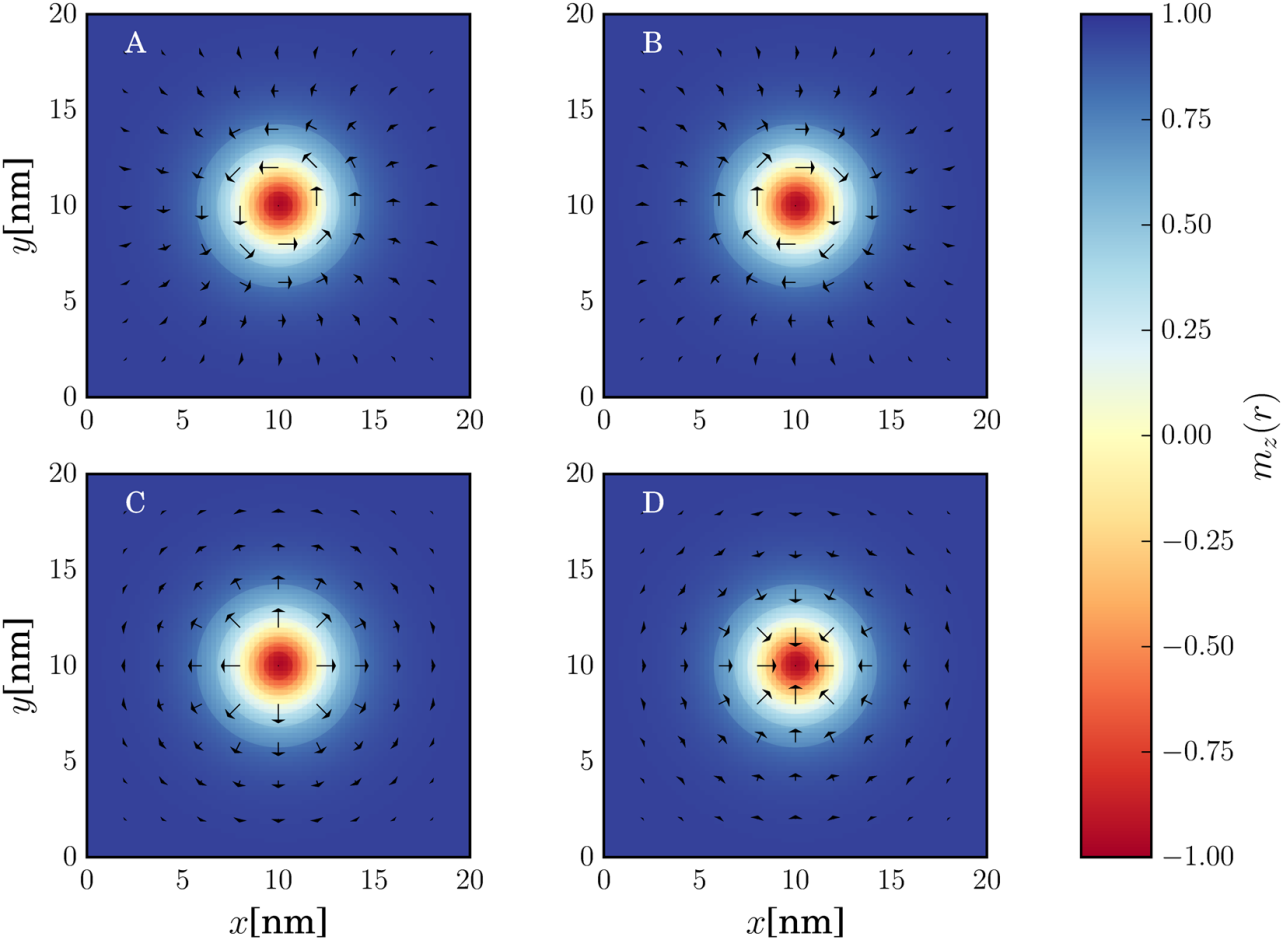
Examples of skyrmion magnetization textures with *r*_*s*_ = *W*/2 on the FM for *L* = *W* = 20 nm. (**A**) Bloch (vortex) skyrmion with *h* = 1, (**B**) Bloch (vortex) skyrmion with *h* = −1, (**C**) Néel (hedgehog) skyrmion with *h* = 1 and (**D**) Néel (hedgehog) skyrmion with *h* = −1. For all skyrmions we have defined *N*_*sk*_ = 1. The colormap refers to the out-of-plane component of the magnetization *m*_*z*_(*r*) with $r=\sqrt{{x}^{2}+{y}^{2}}$. The arrows refer to the in-plane component of the magnetization field. The length of the arrows corresponds to the magnitude of the in-plane component, ${m}_{||}=\sqrt{{m}_{x}^{2}+{m}_{z}^{2}}$ while the direction of the arrow is also the direction of the in-plane magnetization at the point (*x*,* y*).

**Figure 4:**
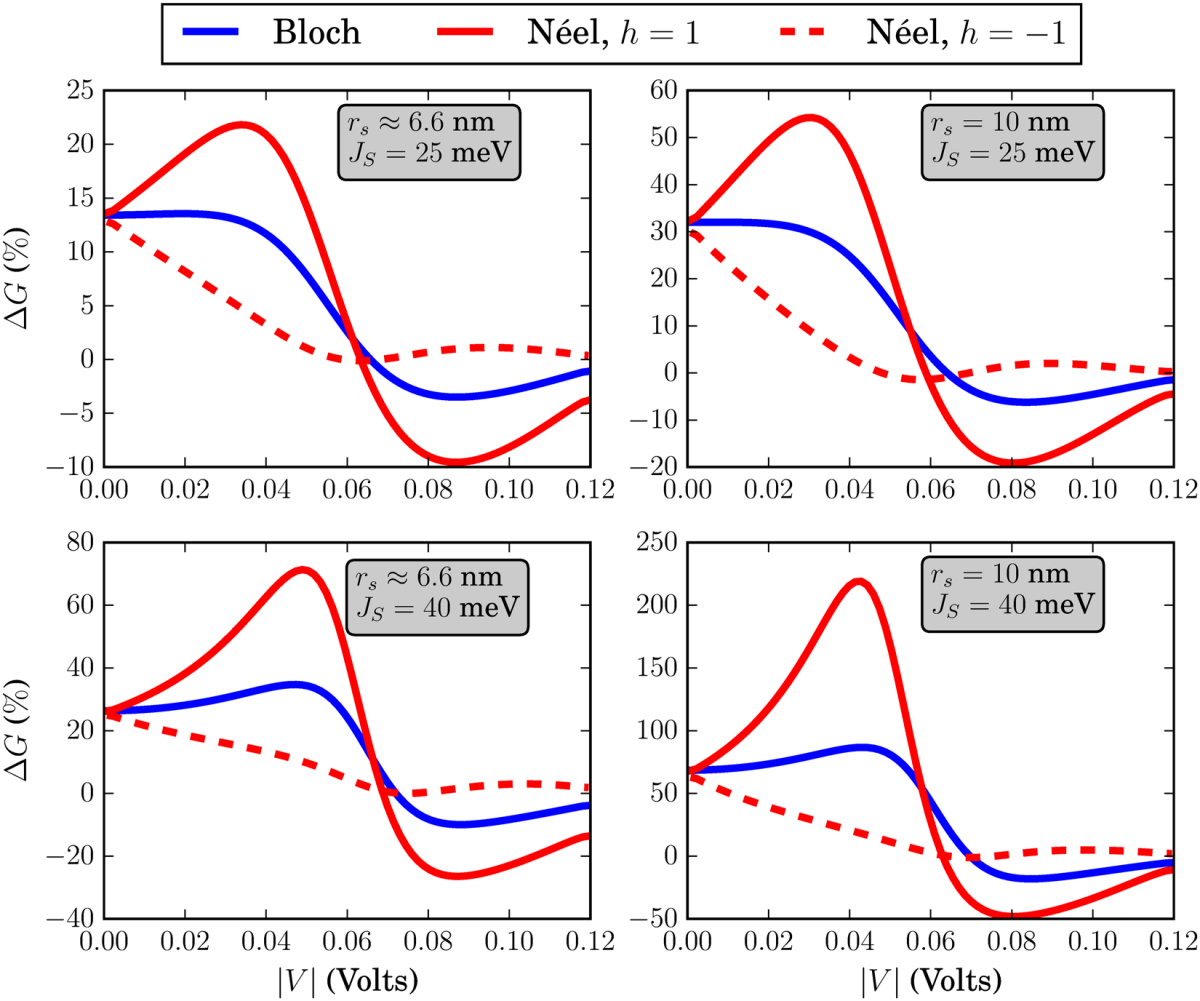
Relative change in conductance Δ*G*, in the low-temperature limit. The FM is of dimensions *L* = *W* = 20 nm. For all skyrmions, *N*_*sk*_ = 1.

**Figure 5:**
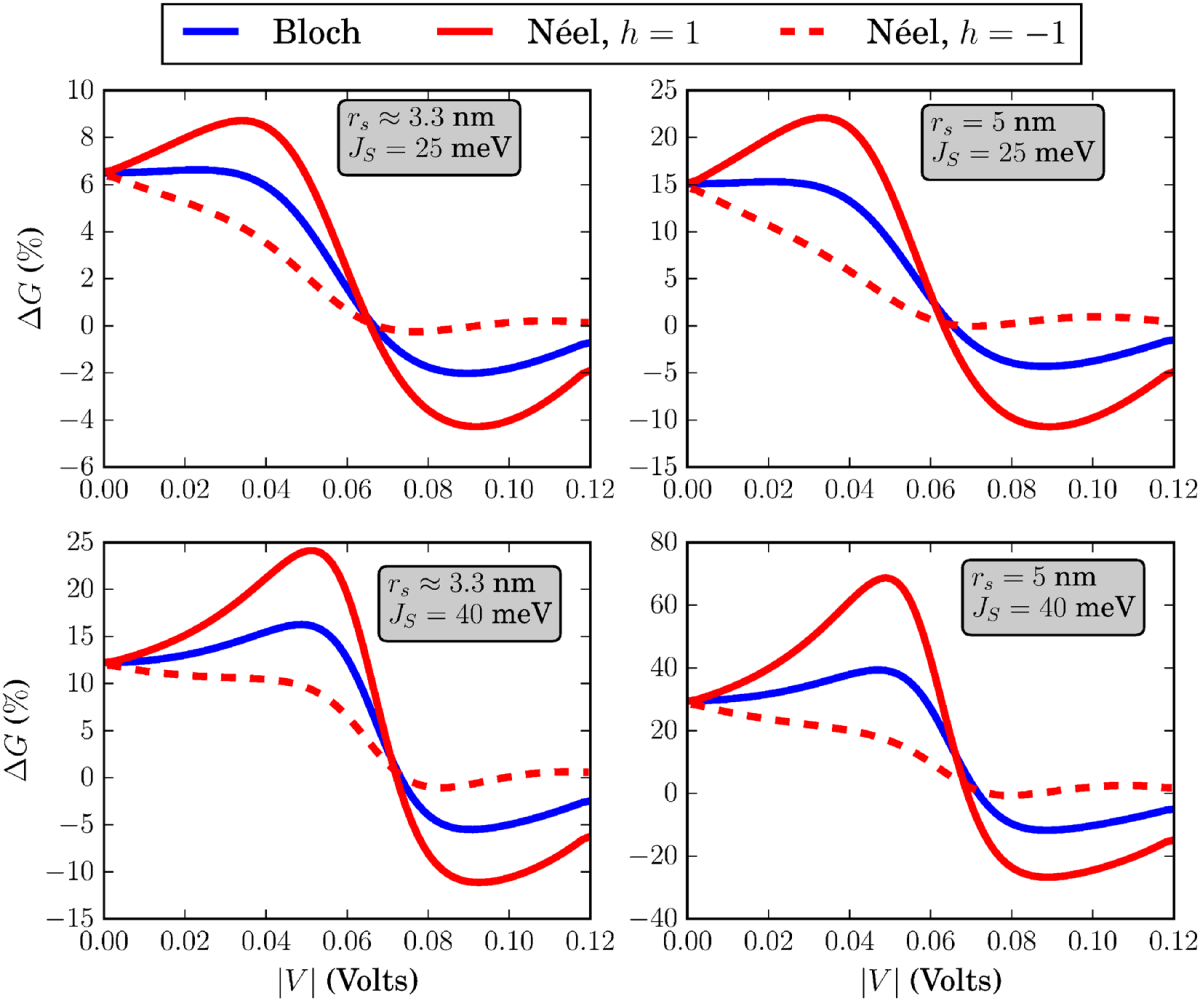
Relative change in conductance Δ*G*, in the low-temperature limit. The FM is of dimensions *L* = 20 nm and *W* = 10 nm. For all skyrmions, *N*_*sk*_ = 1.

**Figure 6:**
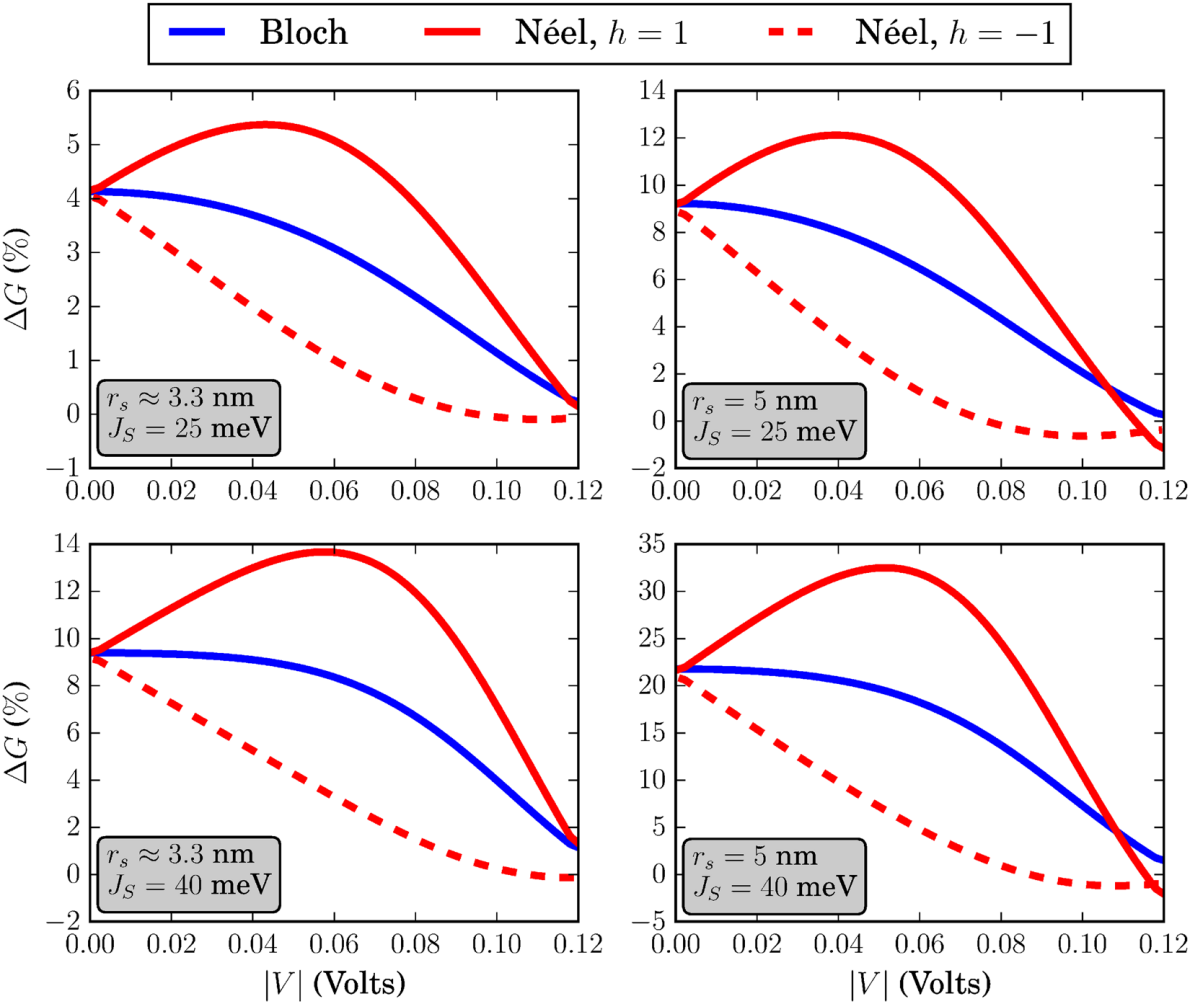
Relative change in conductance Δ*G*, in the low-temperature limit. The FM is of dimensions *L* = *W* = 10 nm. For all skyrmions, *N*_*sk*_ = 1.

**Figure 7:**
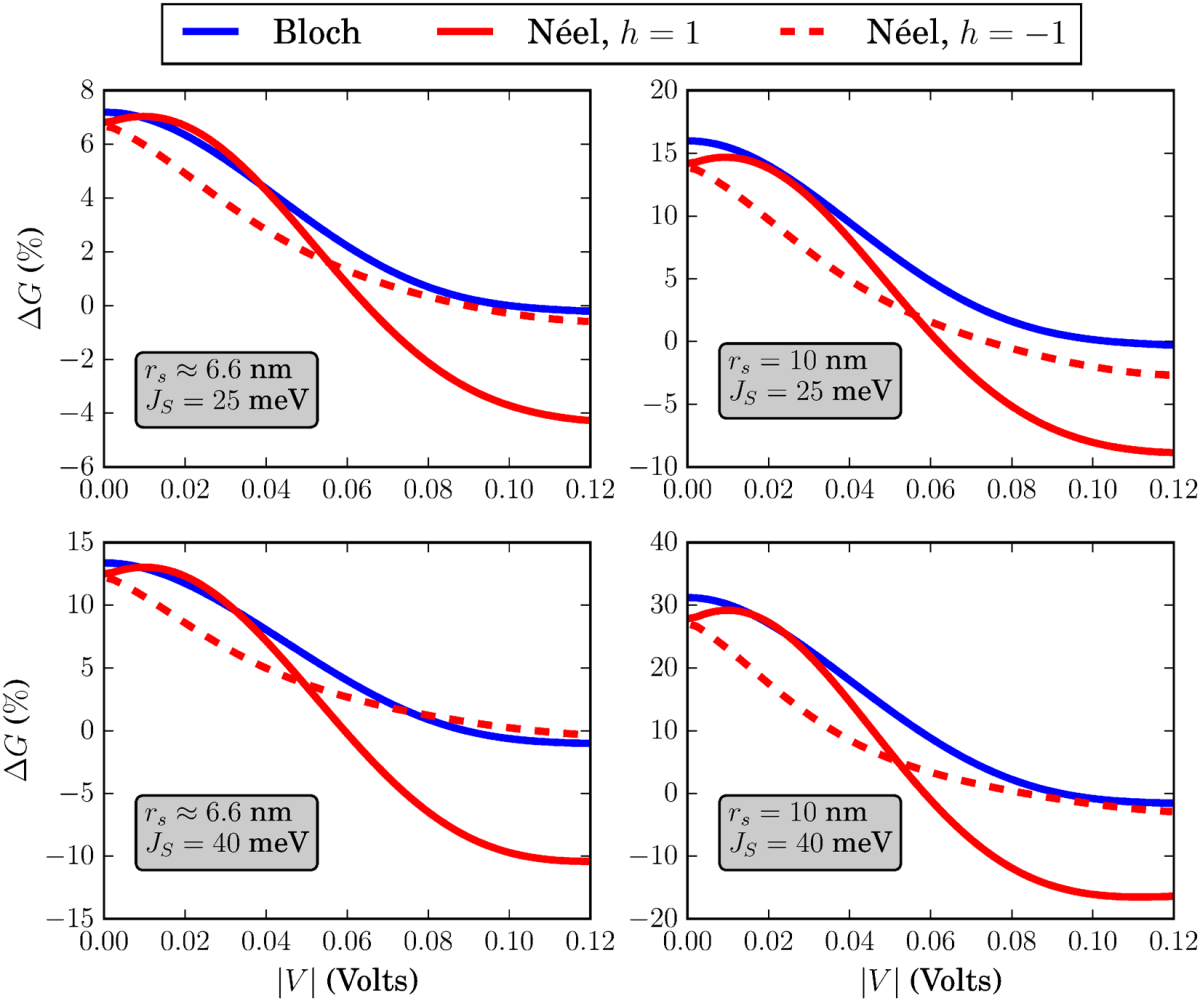
Relative change in conductance Δ*G*, for *k*_*B*_ *T* = 25 meV. The FM is of dimensions *L* = *W* = 20 nm. For all skyrmions, *N*_*sk*_ = 1.

**Figure 8:**
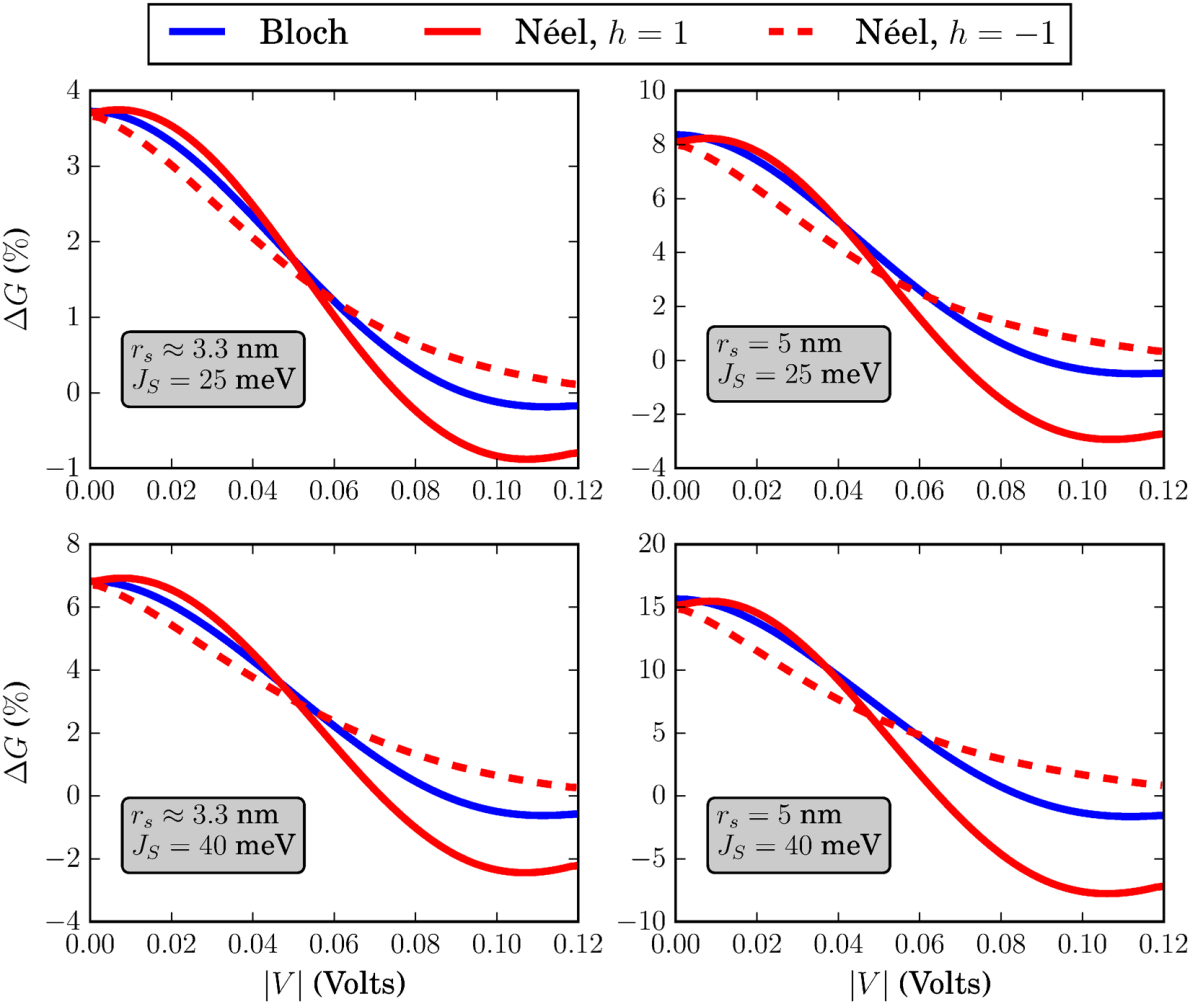
Relative change in conductance Δ*G*, for *k*_*B*_ *T* = 25 meV. The FM is of dimensions *L* = 20 nm and *W* = 10 nm. For all skyrmions, *N*_*sk*_ = 1.

**Figure 9:**
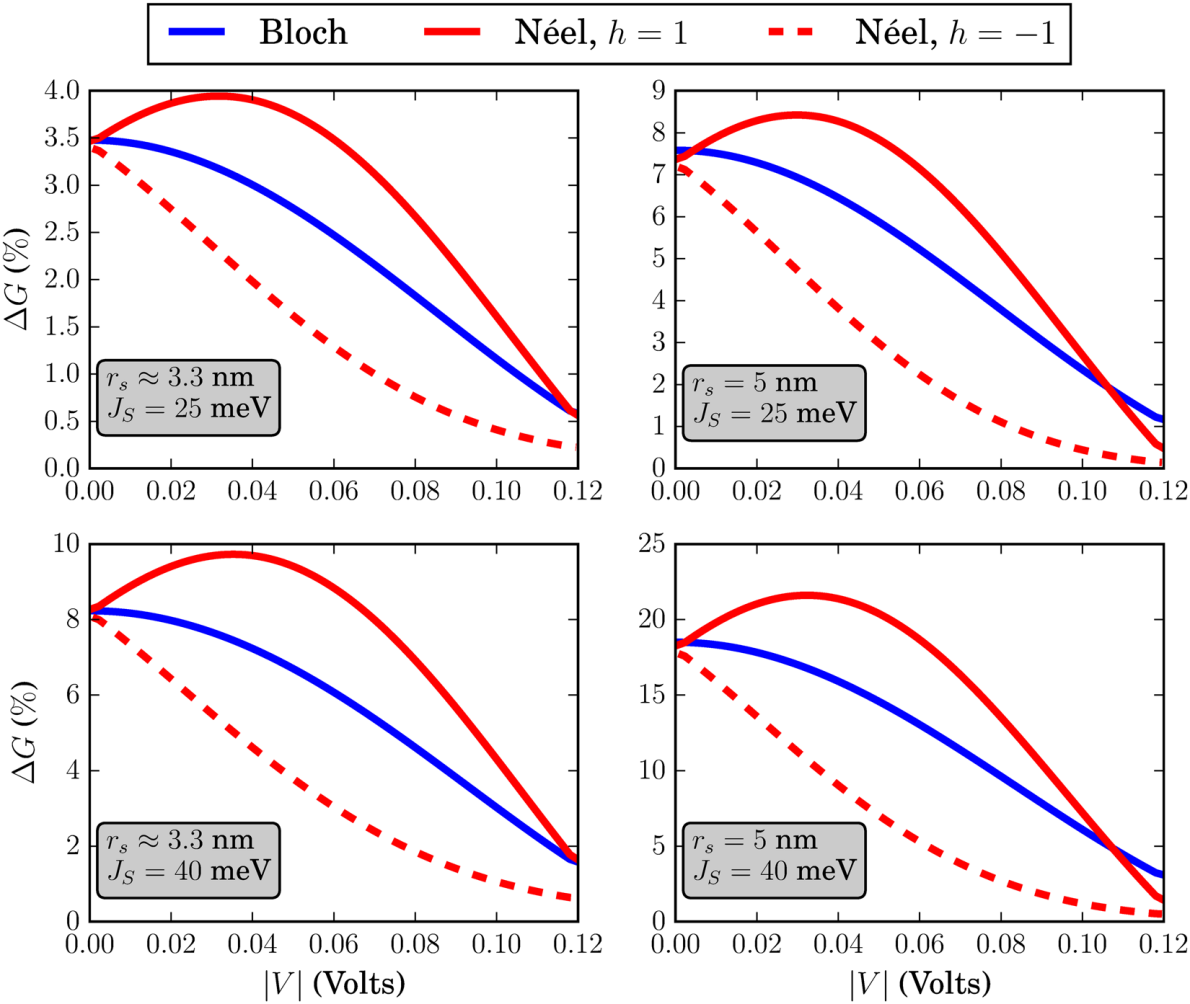
Relative change in conductance Δ*G*, for *k*_*B*_ *T* = 25 meV. The FM is of dimensions *L* = *W* = 10 nm. For all skyrmions, *N*_*sk*_ = 1.

The different skyrmion textures that we have simulated in this work are shown in Fig. 3. Before discussing the effects of each skyrmion type separately, we address some general behavior of the system conductance which can be extracted by qualitatively examining Figs 4–9. There, we plot the relative change in conductance Δ*G* = (*G*_*S*_ − *G*_*R*_)/(*G*_*R*_) × 100% in the low-temperature regime (Figs 4–6) and at room temperature, i.e. for *k*_*B*_ *T* = 25 meV (Figs 7–9), with *G*_*S*_ being the conductance due to the Bloch or Néel skyrmion presence. In each of these figures we fix the dimensions of the FM, to *L* = *W* = 20 nm in Figs 4 and 7, *L* = 20 nm and *W* = 10 nm in Figs 5 and 8 and *L* = *W* = 10 nm in Figs 6 and 9. In the sub-figures we annotate the skyrmion size parameter *r*_*s*_ and interaction strength *J*_*S*_ that we have used for each case. Furthermore, it is useful for the analysis of the results to have the same number of injection modes at the input, for all cases presented in Figs 4–9. More specifically, in this work we focus on single-mode input. Due to the fact that the energy gap between the transverse modes scales as 1/*W*, the largest value of *V*_*in*_ for which a single transverse mode is available at the input is given for *W* = 20 nm. For that case, *V*_*in*_∈[0, 0.12] eV and we restrict the input in this range. We observe that Δ*G* as a function of the input *V*_*in*_ depends on the FM dimensions *L* and *W*, interaction strength *J*_*S*_ and energy *k*_*B*_ *T* as well as on the skyrmion type and size. In the following, we elaborate on each of these factors separately.

To begin with, in the low-temperature regime where *k*_*B*_ *T* → 0, the overall behavior of the curve for Δ*G* in the specified input voltage range strongly depends on the FM length *L*. This can be deduced by noticing the curves in Figs 4 and 5 where *L* = 20 nm and compare these with the corresponding curves in Fig. 6 for which *L* = 10 nm. For *L* = 20 nm, the curves for Δ*G* show an oscillatory-like behavior and in general attain larger values than for the case when *L* = 10 nm. This is an indication that the overall behavior of the conductance for the voltage inputs considered is determined by the length of the FM on the TI, i.e.the length of the energy barrier. Despite the fact that for larger *L* (Figs 4 and 5), the skyrmion area to the total FM area gets smaller, the relative change in conductance Δ*G* is in general higher. Therefore, the effect on conductance, is a combination of both the extent of the FM background magnetization along the longitudinal direction and the specific skyrmion profile. As can be seen in Figs 7 and 8 (*L* = 20 nm) and in Fig. 9 (*L* = 10 nm) at room temperature, the dependence of Δ*G* on the FM length *L* is similar to the low-temperature case, i.e. for smaller *L* we also obtain lower Δ*G* in general.

Furthermore, we observe in Figs 7 and 8 that at room temperature for *k*_*B*_ *T* = 25 meV, the oscillatory behavior is less pronounced for *L* = 20 nm while for *L* = 10 nm (Fig. 9) the shape of the curve of Δ*G* is similar to the one in the low-temperature limit of Fig. 6. In all cases however, the values for Δ*G* are lower for *k*_*B*_ *T* = 25 meV. Nevertheless, we still obtain a finite Δ*G* in RT. We note here that in our simulations, for *k*_*B*_ *T* ≠ 0 we have accounted for a broadened Fermi-Dirac distribution, assuming that the skyrmion texture remains rigid and the TI surface state is still accurately described by Eq. (1). The broadened Fermi-Dirac distribution explains the disappearance of the oscillations of Δ*G* in Figs 7 and 8.

In order to address the effect of the width *W* separately, we notice that changing the width *W* while keeping *L* fixed, does not affect the reference transmission and conductance of a homogeneous magnetic texture without any skyrmion present. It only affects the skyrmion size and the possible transverse modes in the scattering region. Regarding the skyrmion size, in our work we use the parameter *r*_*s*_ to modify the skyrmion radius which is scaled with respect to *W*, namely *r*_*s*_ = *W*/3 or *r*_*s*_ = *W*/2. Consequently, the width *W* affects both the dimensions of the FM as well as the skyrmion size. We consider two widths *W* = 10 nm and *W* = 20 nm resulting in four different values for *r*_*s*_ = {10/3,5,20/3,10}. In order to understand how the relative size of the skyrmion w.r.t. the FM affects Δ*G*, we calculate the ratio *η* of the skyrmion area to the total FM area, i.e.

$$\eta =\frac{\pi {r}_{s}^{2}}{WL}$$

**Figure 10:**
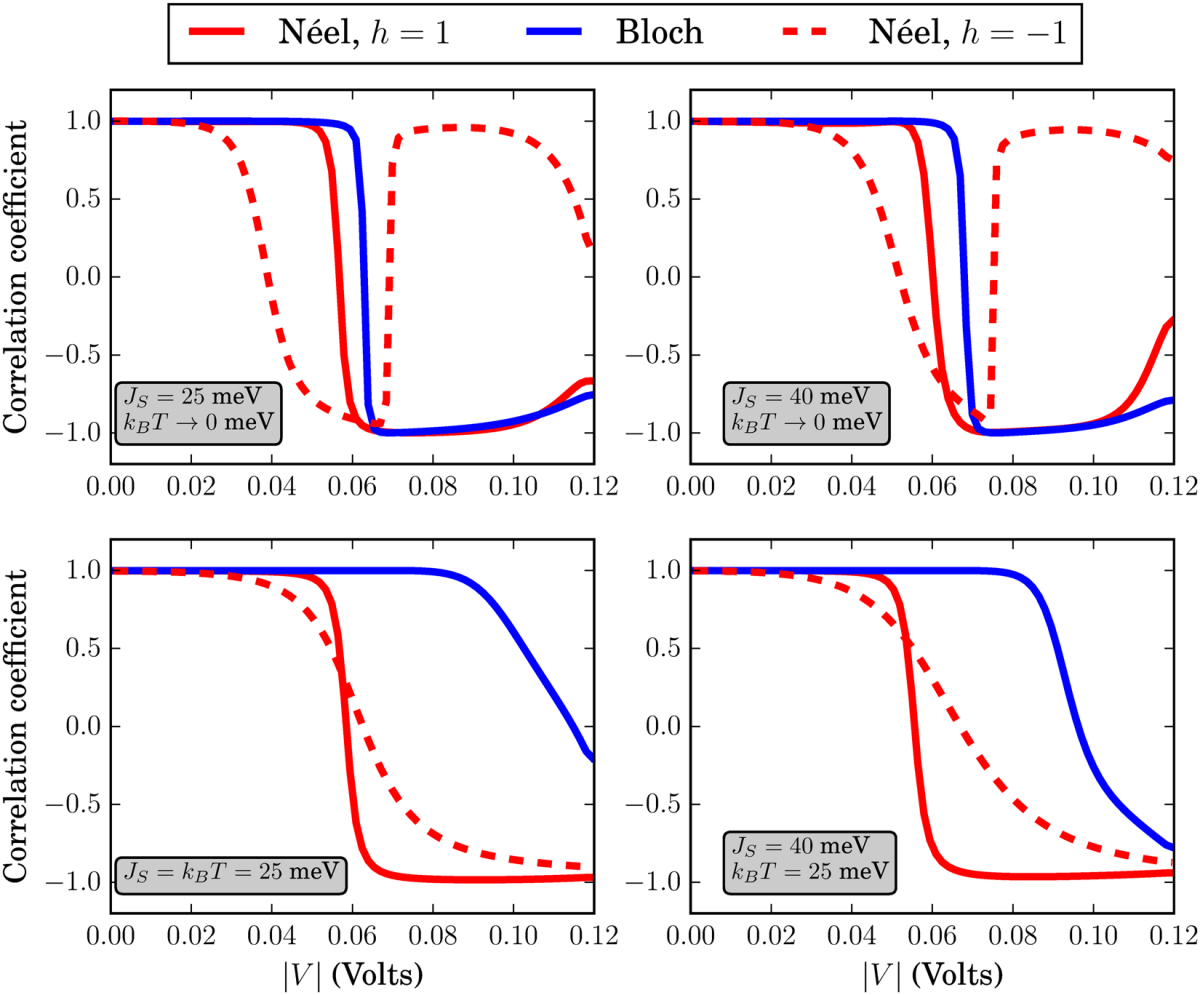
Correlation coefficient between the ratios *η* and Δ*G* of Figs 4 and 5, for Bloch and Néel skyrmions with *h* = ±1. The parameters *J*_*S*_ and *k*_*B*_ *T* are shown in the inset of each figure. The larger the absolute value of the correlation coefficient, the more linearly related are *η* and Δ*G*.

Because we consider two values for *W* while keeping *L* fixed to *L* = 20 nm, this results into four different values for *η* = {*π*/4, *π*/8, *π*/9, *π*/18} corresponding to (*r*_*s*_ = 10, *W* = 20) nm, (*r*_*s*_ = 5, *W* = 10) nm, (*r*_*s*_ = 20/3, *W* = 20) nm and (*r*_*s*_ = 10/3, *W* = 10) nm respectively. Then, we find the correlation coefficient between *η* and Δ*G* for the low-temperature case presented in Figs 4 and 5 and the room temperature cases presented in Figs 7 and 8. A value of the coefficient close to ±1 implies a linear relation between *η* and Δ*G* while a value close to 0 implies no linear relation. In Fig. 10 we plot the Pearson correlation coefficient as a function of the input *V* for Bloch and Néel skyrmions. In the low-temperature limit, i.e. *k*_*B*_ *T* → 0, corresponding to the cases shown in Figs 4 and 5, the relation between *η* and Δ*G* for Néel skyrmions with *h* = 1 and Bloch skyrmions is linear for almost every input *V*. Around *V* ≈ 60 meV, we have an abrupt change of this linear relation from positive to negative. A positive correlation coefficient here means that as the relative skyrmion area w.r.t. the FM area or *η*, increases, Δ*G* increases as well, while with a negative correlation coefficient, as *η* increases, Δ*G* decreases. For high input *V* the relation becomes less linear since the absolute value of the coefficient gets smaller. This is due to the fact that for high input *V*, Δ*G* → 0, because the interaction with the magnetization texture becomes less effective. For Néel skyrmions with *h* = −1, the relation is only linear for a narrower input range than the case of Bloch or Néel skyrmions with *h* = 1. For *k*_*B*_ *T* = 25 meV, the linear relation still holds for Néel skyrmions with *h* = 1 with an abrupt change at *V* ≈ 60 meV. Furthermore, for Néel skyrmions with *h* = −1 a linear relation is obtained but the change of sign occurs in a wider input range meaning that there is a finite range of input values for which the relation is not linear. Finally, *η* ∝ Δ*G* also for Bloch skyrmions with the relation becoming less linear for higher inputs and *J*_*S*_ = 25 meV, while it changes sign when the interaction strength is increased.

Finally, the effect of the interaction strength *J*_*S*_ is to enhance or reduce the effect of the magnetization texture on the TI surface state. Increasing the value of *J*_*S*_, leads to a wider input range for which there is appreciable interaction with the FM texture. Additionally, the deviation from the trivial texture is larger in this case and this is illustrated by the fact that Δ*G* in general, is larger for a higher value of *J*_*S*_. This means that the modification of **m**(*x*, *y*) by the skyrmion, is felt more strongly by the electron on the TI surface.

### Bloch (vortex) skyrmion

**Figure 11:**
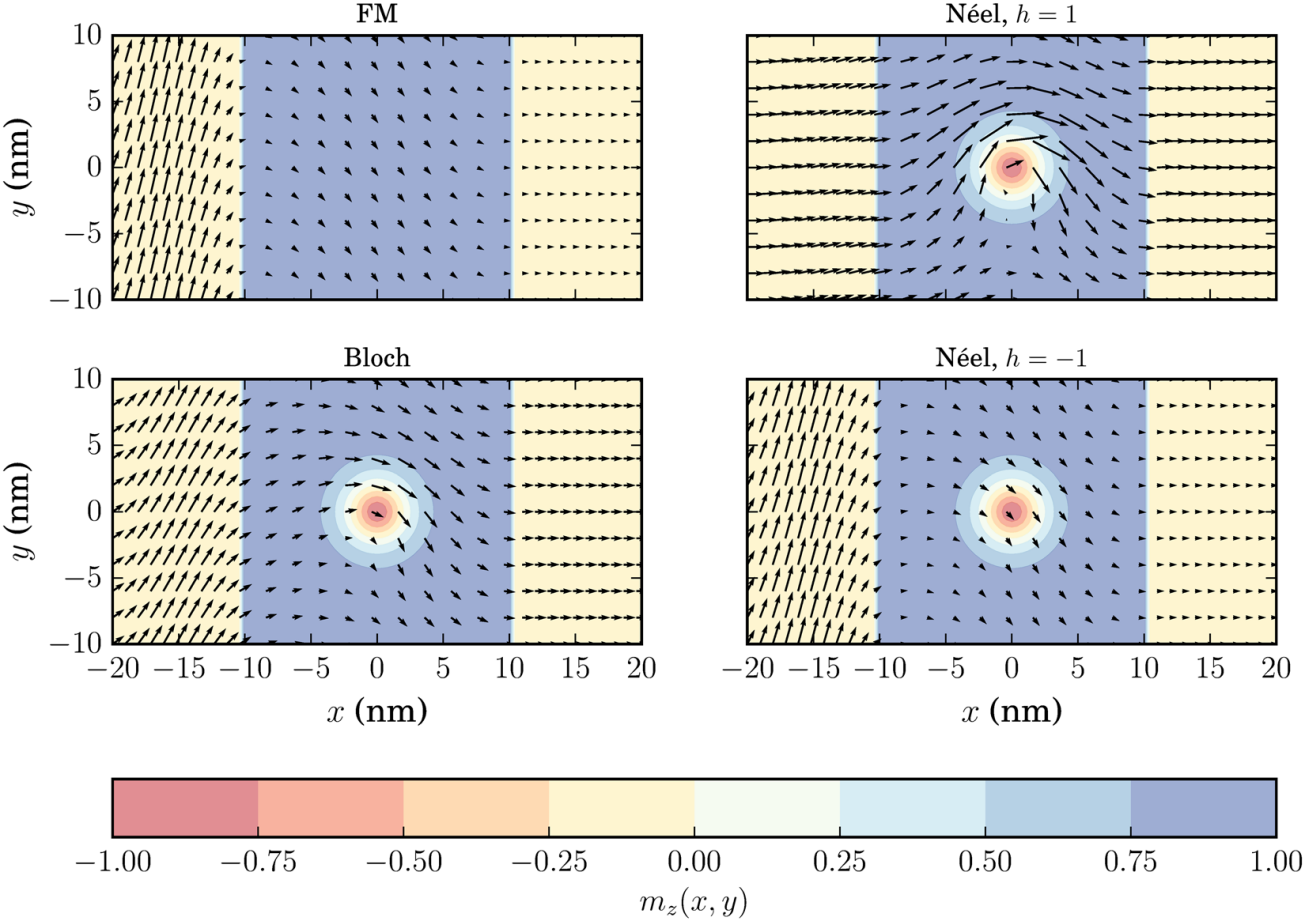
Steady state probability current densities for a homogeneous texture (top left), Bloch skyrmion (bottom left), Néel skyrmion with *h* = 1 (top right) and Néel skyrmion with *h* = −1 (bottom right). For all cases, *L* = W = 20 nm, *J*_*S*_ = 40 meV, *r*_*s*_ = W/2 = 10 nm and $|{V}|$ = 50 mV. The length of the arrows corresponds to the magnitude of the current and the direction of the current coincides with the direction of the arrows. The colormap in the background represents *m*_*z*_(x, y).

For the Bloch skyrmion texture, shown in Fig. 3A and B, the relative conductance change is shown by the blue line in Figs 4–6. For this skyrmion type interacting with the TI surface, it can be shown that the in-plane magnetization components can be gauged away^33,43^. Therefore, the only modifcation of the reference texture due to the skyrmion presence is attributed to the perpendicular component *m*_*z*_(*x*,*y*) which acts as a space-dependent mass term in the Dirac Eq. (1). This term alters the energy barrier of the uniform FM background magnetization to a non-uniform energy barrier. Thus, scattering of the wavefunction is expected not only at the interfaces with the lead regions, but also throughout the FM region. This additional scattering results into the modification of the transmission probabilty and therefore of the conductance of the system. To better illustrate the effect of the non-uniform *m*_*z*_ term, we plot in Fig. 11 the probability current density ***j***(*x*,*y*) for the trivial (top left subfigure of Fig. 11) and Bloch skyrmion texture (bottom left subfigure of Fig. 11). The probability current has been calculated taking into account the total wavefunction which results from the solution of the scattering problem. Therefore, the solution contains incident, reflected and transmitted parts. For finite reflection amplitude in the leads, as is shown in the methods section, the resulting probability current density contains components both along $\hat{{\bf{x}}}$ and $\hat{{\bf{y}}}$. This is the reason why the probablity current direction at the input leads (*x* < −10 nm) in Fig. 11 is not purely along $\hat{{\bf{x}}}$. For the case presented in this figure, *G*_*S*_/*G*_0_ ≈ 0.65 and *G*_*R*_/*G*_0_ ≈ 0.36. It is evident that the effect of the non-uniform *m*_*z*_ component is to induce such scattering to the wavefunction inside the FM region so that the *j*_*x*_ component is enhanced. Since we have defined the quantities of interest (transmission and conductance) through *j*_*x*_, it is straightforward to prove that this enhancement leads to an increase of the conductance. Although this is vald for this input voltage *V*, it can happen that for other input values, the *j*_*x*_ component is diminished. This is due to the fact that the scattering inside the FM region strongly depends on the input energy *qV*, which remains constant as we have elastic scattering.

Another interesting observation one can make from Fig. 11 is the fact that the skyrmion induces non-symmetric scattering as can be seen from the asymmetry of the probability current density around the *y* = 0 line. This is explained as follows: the skyrmion texture constitutes a curved boundary between regions with different values of the mass term. For a straight boundary, the reflection at the interface is the same for every *y*, but it depends on the incident angle *θ*_*in*_ = *arctan*(*k*_*x*_/*k*_*y*_). Since we are dealing with a curved boundary, at each point along the boundary this angle is different and thus we expect non-uniform scattering along $\hat{{\bf{y}}}$. This non-symmetric scattering is also shown analytically in a recent work^42^.

### Néel (hedgehog) skyrmion

The Néel skyrmion texture is shown in Fig. 3C and D and the corresponding Δ*G* is depicted by the red solid (positive helicity) and dashed (negative helicity) lines in Figs 4–6. For positive helicity, *h* = 1, Δ*G* can be much larger than the corresponding one for Bloch skyrmions, while for negative helicity, *h* = −1, Δ*G* attains much lower values. This behavior is explained by the specific structure of the in-plane magentization components interacting with the spin-momentum locked surface states. Contrary to the Bloch skyrmion, for which the in-plane components are gauged away, for Néel skyrmions they cannot, and they act as a local emergnt field along $\hat{{\bf{z}}}$ with magnitude ${B}_{\parallel }^{(e)}=\nabla \cdot {{\bf{m}}}_{\parallel }$ ^33,43^. The direction of the in-plane components (helicity) determines the sign of the emergent field and enhances or decreases the effect of the out-of-plane magnetization component thus increasing or decreasing Δ*G*. This is illustrated in the probability current density plot for the Néel skyrmions in Fig. 11. For a Néel skyrmion with *h* = 1 (top right subfigure of Fig. 11), *G*_*S*_/*G*_0_ ≈ 0.97, while for a Néel skyrmion with *h* = −1 (bottom right subfigure of Fig. 11), *G*_*S*_/*G*_0_ ≈ 0.4. The Bloch skyrmion, essentially boosts the *j*_*x*_ component of the proabibility current density with respect to the reference texture. For a Néel skyrmion with positive helicity, i.e. *h* = 1, this effect is more pronounced as can be seen in the top right vector plot in Fig. 11. This is attributed to the spin-momentum locking mechanism on the TI surface. The specific structure of the in-plane components of the Néel skyrmion with positive helicity (Fig. 3C) boosts the effect of the out-of-plane component by scattering surface state wavefunction in such a way that the probability current structure of a Bloch skyrmion is enhanced. This does not mean however that for every input voltage, the conductance for a Néel skyrmion is always higher than that of a Bloch skyrmion, as the overall result is a combination of the specific scattering experienced by the wavefunction in the FM region.

On the other hand, reversing the helicity of the Néel skyrmion (bottom right subfigure of Fig. 11), changes the sign of the emergent field and the scattering induced tries to cancel out the effect from the mass term. In that case, the skyrmion cannot be efficiently distinguished from the trivial magnetization background when no skyrmion is present. This is attributed to the spin-momentum locking mechanism of the TI surface and the specific structure of the in-plane components of the skyrmion magnetization texture. In all cases, the surface electrons try to align their spin in parallel with that of the in-plane skyrmion components in order to minimize the energy as can be seen from Eq. (1). Through the spin-momentum locking mechanism however, when a Néel skyrmion wih negative heliciy is present, the particular spin alignment is connected with momenta that give a probability current density which opposes the one that is generated by the scattering of the wavefunction from the out-of-plane magnetization component *m*_*z*_. As a result, the two scattering mechanisms from the in-plane and out-of-plane skyrmion components almost cancel out leaving the transmission probability and thus the conductance approximately unaffected compared to the reference case of a uniformly magnetized FM without any skyrmion present.

Since the effect of the Néel skyrmion depends on the combination of the type of scattering induced by the out-of-plane and in-plane components, changing the sign of either of them renders the Néel skyrmion transparent or strongly distinguishable from the FM background. In the above we explained that in order to change the scattering from the in-plane components, the reversal of the skyrmion helicity is necessary. Similarly, if we make the skyrmion topological number negative (*N*_*sk*_ = −1), the scattering from the out-of-plane component is also the opposite. Therefore, in that case, a skyrmion with negative helicity will look like a skyrmion with positive helicity and positive topological number. Thus, we conclude that a Néel skyrmion can be a sufficiently distinct magnetization texture iff sgn(*N*_*sk*_) = sgn(*h*).

## Discussion

In this work, we have investigated the interaction of the TI surface state with Néel and Bloch skyrmion magnetization textures. By numerically solving the transport problem, we are able to calculate the conductance of the system in both cases and compare it with the reference conductance of a uniform FM magnetization background without any skyrmion present. For both skyrmion types, we have shown that there can be a modification of the conductance that depends on several parameters including the dimensions of the skyrmion and the FM, the skyrmion type and the interaction strength.

Regarding the FM dimensions, the length *L* will affect mostly the reference conductance while the width of the system affects *η* being the relative skyrmion area to the total FM area. With *L* fixed, the change in conductance Δ*G* is proportional to *η* for every skyrmion type up to *V* ≈ 60 meV as illustrated by the correlation coefficient in Fig. 10. For higher input values of *V*, depending on the skyrmion type, *J*_*S*_ and *k*_*B*_ *T*, the strength of the linearity between *η* and Δ*G* can change.

Regarding the skyrmion type, the emergent gauge field of the Néel skyrmion shows a detrimental effect on the conductance of the system as it can either enhance (*h* = 1) or counteract (*h* = −1) the effect of the out-of-plane component *m*_*z*_, rendering the Néel skyrmion either strongly distinguishable (positive helicity, *h* = 1) or identical (negative helicity, *h* = −1) to the FM background.

**Figure 12:**
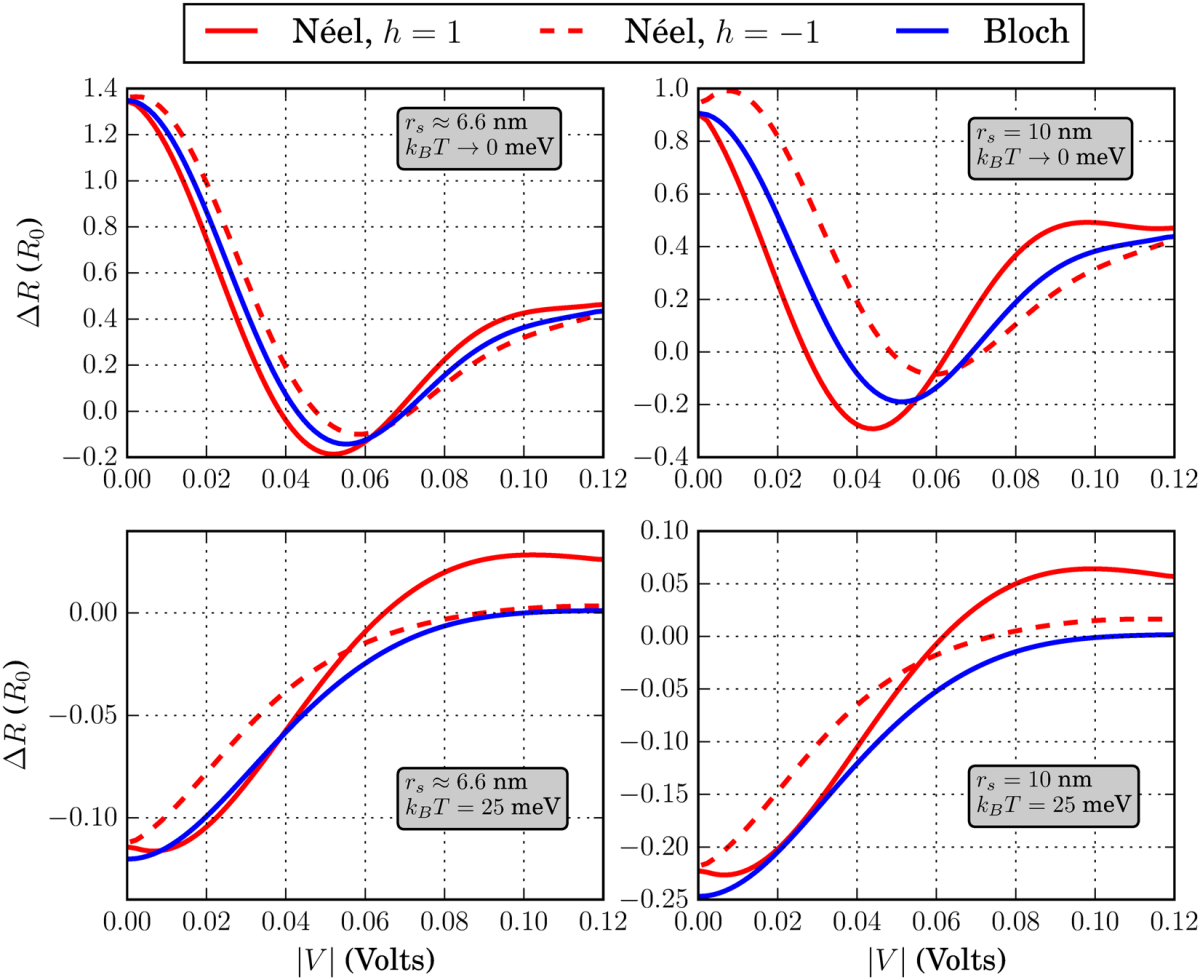
Resistance change Δ*R* in units of *R*_0_ = *h*/*q* ^2^ for *L* = *W* = 20 nm. The skyrmion sizes and *k*_*B*_ *T* are shown in the inset of each figure. For all cases, *J*_*S*_ = 25 meV and *N*_*sk*_ = 1

**Figure 13:**
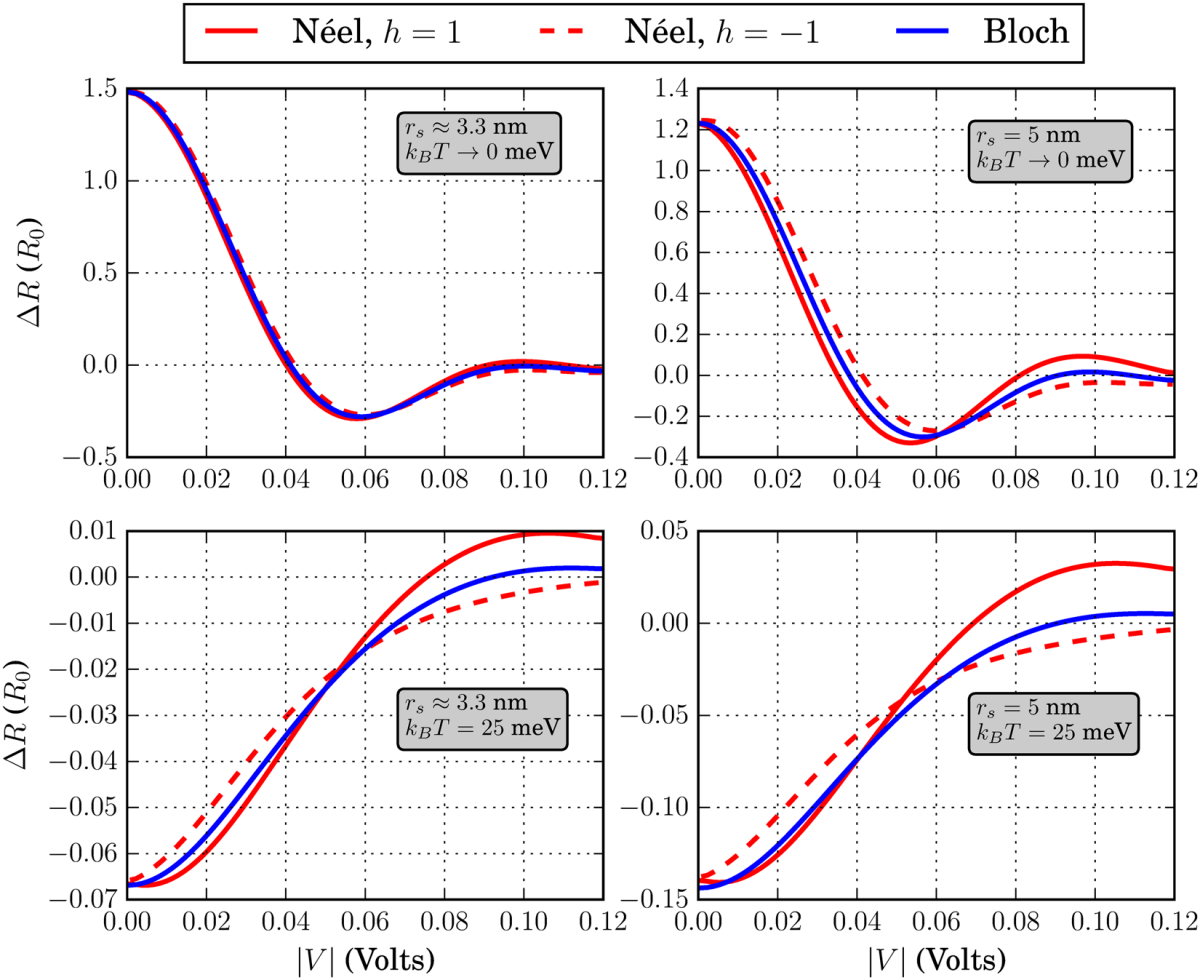
Resistance change Δ*R* in units of *R*_0_ = *h*/*q* ^2^ for *L* = 20 nm and *W* = 10 nm. The skyrmion sizes and *k*_*B*_ *T* are shown in the inset of each figure. For all cases, *J*_*S*_ = 25 meV and *N*_*sk*_ = 1

**Figure 14:**
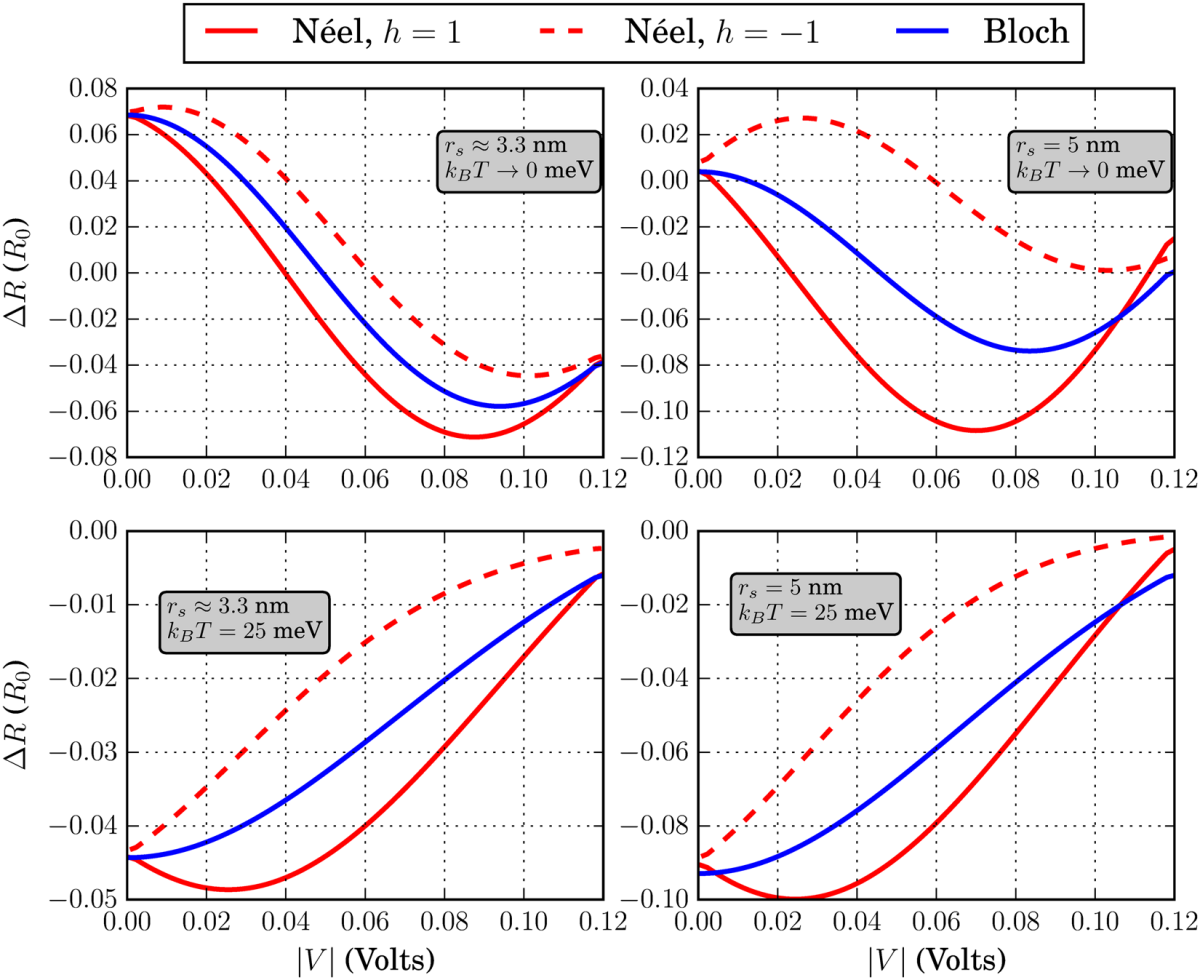
Resistance change Δ*R* in units of *R*_0_ = *h*/*q* ^2^ for *L* = *W* = 10 nm. The skyrmion sizes and *k*_*B*_ *T* are shown in the inset of each figure. For all cases, *J*_*S*_ = 25 meV and *N*_*sk*_ = 1

To demonstrate the fact that the skyrmion presence can give a distinct electronic signal, we plot in Figs 12–14 the resistance change Δ*R* = *R*_*S*_ − *R*_*R*_ = 1/*G*_*S*_ − 1/*G*_*R*_ in units of *R*_0_ = *h*/*q* ^2^. In each of these figures, we fix *L* and *W* and vary the skyrmion radius and *k*_*B*_ *T*. For all cases, *J*_*S*_ = 25 meV. It is shown that even at room temperature, the skyrmion presence can induce a change in the resistance up to 0.25 *R*_0_ ≈ 6 k Ω namely in the case *L* = *W* = 20 nm and for *r*_*s*_ = 10 nm and *J*_*S*_ = 40 meV. In the low-temperature regime, Δ*R* can be of the order of 10^4^ Ω. Increasing the interaction strength *J*_*S*_ will enhance Δ*R* in both temperature regimes. Increasing the relative skyrmion area w.r.t. the FM area, quantified by *η*, also increases the resistance change Δ*R*.

Finally, we compare our skyrmion electrical detector with some recently proposed schemes. First, our scheme requires current in-plane and as a results does not involve more complex structures as the ones required for non-collinear magnetoresistance^24,27,28^. While the main advantage of the latter is the ability to detect lattice constant - sized skyrmions, our method is more applicable for skyrmions with sizes larger than a few lattice constants since we use a continuum approximation to describe the skyrmion texture. In^25,26^, the authors use conduction electrons in plane that strongly interact with the skyrmion texture. The emergent magnetic field from the skyrmion induces a Berry phase and therefore a THE signal is obtained. The sign of the Berry phase depends on the spin of the electron^54,55^ and as a result, the THE signal will be weaker if there is a comparable number of carriers occuppying both spin bands. In our case however we do not impose a strong coupling condition and the unique nature of the TI surface ensures that the incident electrons occupy a specific spin band. The THE schemes^25,26^ have the advantage of being robust to elastic and inelastic scattering and to temperature variations. In our work, ideally, the only scattering that can occur is due to magnetic impurities, which we assume that we can efficiently control, while we have shown that even at room temperature we can still obtain a detectable signal for the skyrmion presence, under the assumption that the surface state is still described by Eq. (1) and the skyrmion texture is robust.

## Methods

### Skyrmion texture

The magnetization texture of the FM is a three-dimensional vector parametrized by the angles Θ(*r*) and Φ(*ϕ*), as shown in Eq. (2). In polar coordinates, *x* = *rcos*(*ϕ*) and *y* = *rsin*(*ϕ*).

2 $${\bf{m}}=(\sin \,{\rm{\Theta }}(r)\cos \,{\rm{\Phi }}(\varphi ),\,\sin \,{\rm{\Theta }}(r)\sin \,{\rm{\Phi }}(\varphi ),\,\cos \,{\rm{\Theta }}(r))$$

For the trivial ferromagnetic texture, Θ(*r*) = 0. For the skyrmion texture, we use a model for Θ(*r*) derived from Lagrangian minimization as in our previous work^33^,

$${\rm{\Theta }}(r)=\pi {e}^{-br}\,,\,b=\frac{1}{{r}_{s}}\,\mathrm{log}({10}^{-1}/\pi )$$

The skyrmion radius is not a very well defined parameter and we use *r*_*s*_ as a parameter to denote the radius where ${m}_{\parallel }/{m}_{z}=0.1$. The angle Φ(*ϕ*) = *ϕ* + *γ* where *γ* is a phase that modifies the in-plane component helicity *h* as follows:

$$\gamma =\{\begin{array}{ll}0, & {\rm{hedgehog}}\,{\rm{skyrmion}}\,(N{\acute{e}}\mathrm{el})\,{\rm{with}}\,h=+1\\ \pi , & {\rm{hedgehog}}\,{\rm{skyrmion}}\,(N{\acute{e}}\mathrm{el})\,{\rm{with}}\,h=-1\\ \pi \mathrm{/2}, & {\rm{vortex}}\,{\rm{skyrmion}}\,(\mathrm{Bloch})\,{\rm{with}}\,h=+1\\ 3\pi \mathrm{/2}, & {\rm{vortex}}\,{\rm{skyrmion}}\,(\mathrm{Bloch})\,{\rm{with}}\,h=-1\end{array}$$

The skyrmion topological number is then defined as follows:

3 $$\begin{array}{ll}{N}_{sk} & =-\frac{1}{4\pi }{\int }_{0}^{\infty }r{\rm{d}}r{\int }_{0}^{2\pi }{\rm{d}}\varphi \,{\bf{m}}\cdot (\frac{\partial {\bf{m}}}{\partial x}\times \frac{\partial {\bf{m}}}{\partial y})\\  & =-\frac{1}{2}{[-{m}_{z}(r)]}_{r=0}^{r=\infty }\end{array}$$

Consequently, the skyrmion number in our model is defined by the boundaries we set on the out-of-plane magnetization *m*_*z*_.

### Transmission problem - Analytical Solutions

The transmission problem can be solved analytically when a unifom magnetization along $\hat{{\bf{z}}}$ is present:

4 $${\bf{m}}(x,y)=\{\begin{array}{cc}\hat{{\bf{z}}} & |x|\le L\\ 0 & |x| > L\end{array}$$

For this case,

5 $$H={v}_{F}{({\bf{p}}\times {\boldsymbol{\sigma }})}_{\hat{{\bf{z}}}}-{J}_{S}{\bf{m}}\cdot {\boldsymbol{\sigma }}$$

The time-independent Schrödinger equation *H*Ψ = *E*Ψ yields two coupled differential equations for the spinor components of $${\rm{\Psi }}={(\begin{array}{cc}{\psi }_{a} & {\psi }_{b}\end{array})}^{T}$$:

6a $${v}_{F}\hslash (-\frac{\partial }{\partial x}+i\frac{\partial }{\partial y}){\psi }_{b}=(E+{J}_{S}{m}_{z}){\psi }_{a}$$

6b $${v}_{F}\hslash (\frac{\partial }{\partial x}+i\frac{\partial }{\partial y}){\psi }_{a}=(E-{J}_{S}{m}_{z}){\psi }_{b}$$

Because the magnetization texture has translational invariance along $\hat{{\bf{y}}}$, we have ${\rm{\Psi }} \sim {e}^{i{k}_{y,n}y}$ with *k*_*y*,*n*_ = 2*nπ*/*W*. Then, decoupling the two differential equations yields one second-order differential equation for one of the spinor components. Here, we choose *ψ*_*a*_:

7 $$-\frac{{{\rm{d}}}^{2}}{{\rm{d}}{x}^{2}}{\psi }_{a}(x)=(\frac{{E}^{2}-{J}_{S}^{2}{m}_{z}^{2}}{{({v}_{F}\hslash )}^{2}}-{k}_{y,n}^{2}){\psi }_{a}(x)$$

which has solutions of the form ${\psi }_{a} \sim {e}^{i{k}_{x}x}$ where

$${k}_{x}=\sqrt{(\frac{{E}^{2}-{J}_{S}^{2}{m}_{z}^{2}}{{({v}_{F}\hslash )}^{2}}-{k}_{y,n}^{2})}$$

For the transmission problem we divide the problem into three regions as following: for region 1, *x* ≤ − *L*, for region 2 *x* ≤ *L* and for region 3, *x* ≥ *L*. In region 1 we have an incident and a reflected wave, in region 2 we have one left-moving and one right-moving waves while in region 3 we set a boundary condition having a transmitted wave.

Consequently,

8a $${\psi }_{a}^{\mathrm{(1)}} \sim {e}^{i{k}_{x\mathrm{,1}}x}+r{e}^{-i{k}_{x\mathrm{,1}}x}$$

8b $${\psi }_{b}^{\mathrm{(1)}} \sim {e}^{i{k}_{x\mathrm{,1}}x}{e}^{i(\pi -{\theta }_{n})}-r{e}^{-i{k}_{x\mathrm{,1}}x}{e}^{i{\theta }_{n}}$$

8c $${\psi }_{a}^{\mathrm{(2)}} \sim A{e}^{i{k}_{x\mathrm{,2}}x}+B{e}^{-i{k}_{x\mathrm{,2}}x}$$

8d $${\psi }_{b}^{\mathrm{(2)}} \sim A{z}_{+}{e}^{i{k}_{x\mathrm{,2}}x}-B{z}_{-}{e}^{-i{k}_{x\mathrm{,2}}x}$$

8e $${\psi }_{a}^{\mathrm{(3)}} \sim t{e}^{i{k}_{x\mathrm{,1}}x}$$

8f $${\psi }_{b}^{\mathrm{(3)}} \sim t{e}^{i{k}_{x\mathrm{,1}}x}{e}^{i(\pi -{\theta }_{n})}$$

where $r\in {\mathbb{C}}$ is the reflection amplitude, $t\in {\mathbb{C}}$ is the transmission amplitude, $A,B\in {\mathbb{C}}$ and

$${k}_{x\mathrm{,1}}=\frac{1}{{v}_{F}\hslash }\sqrt{{E}^{2}-{({v}_{F}\hslash {k}_{y,n})}^{2}}\in {\mathbb{R}}$$

$${k}_{x\mathrm{,2}}=\frac{1}{{v}_{F}\hslash }\sqrt{{E}^{2}-{J}_{S}^{2}-{({v}_{F}\hslash {k}_{y,n})}^{2}}\in {\mathbb{C}}$$

$${\theta }_{n}=\arctan (\frac{{k}_{x\mathrm{,1}}}{{k}_{y,n}})$$

$${z}_{\pm }=\frac{{v}_{F}\hslash }{E-{J}_{S}}(i{k}_{x\mathrm{,2}}\mp {k}_{y,n})\mathrm{.}$$

Imposing the continuity of the spinor components at *x* = 0 and *x* = *L*, we construct the linear problem **Ax** = **b** where $${\bf{x}}={(\begin{array}{cccc}r & A & B & t\end{array})}^{T}$$. We solve this problem and obtain an analytical expression for the transmission amplitude as following:

9 $$t=\frac{{e}^{-i{k}_{x\mathrm{,1}}L}({z}_{+}+{z}_{-})\sin ({\theta }_{n})}{(1-{z}_{+}{z}_{-})\sin ({k}_{x\mathrm{,2}}L)-{z}_{-}\,\sin ({k}_{x\mathrm{,2}}L-{\theta }_{n})+{z}_{+}\,\sin ({k}_{x\mathrm{,2}}L+{\theta }_{n})}$$

Consequently, the transmission amplitude (as well as *A*,*B* and *r*), will depend on the incident energy *E*, the transverse mode *n*, the interaction strength *J*_*S*_, and the FM length *L*. For given *J*_*S*_, *L* and *n*, it shows an oscillating behavior as a function of the input energy *E*. From the zeros of the reflection amplitude, we can extract the resonant points for each transverse mode *n*. For *n* = 0, which is the only mode available for low input voltage, these points are found for $\sin ({k}_{x\mathrm{,2}}L)=0\Rightarrow \frac{L}{{v}_{F}\hslash }\sqrt{{E}^{2}-{J}_{S}^{2}}L=2n\pi$ which is independent of the width *W*.

### Probability current density

For the TI hamiltonian (1), the probablity current density is

$${\bf{j}}={v}_{F}(\langle {\sigma }_{y}\rangle \hat{{\bf{x}}}-\langle {\sigma }_{x}\rangle \hat{{\bf{y}}})$$

which is a consequence of the spin-momentum locking mechanism of the TI surface. For a general **m**(*x*), the transverse wave-number is a good quantum number and we can write the wavefunction in each constant-magnetization region as follows:

10 $$\begin{array}{ll}{\psi }_{a} & =\,A{e}^{i{k}_{x}x}+B{e}^{-i{k}_{x}x}\\ {\psi }_{b} & =\,A{z}_{+}{e}^{i{k}_{x}x}-B{z}_{-}{e}^{-i{k}_{x}x}\end{array}$$

The probability current density then obtains the following form:

11a $${j}_{x}=2{v}_{F}{\rm{Im}}({\psi }_{a}^{\ast }{\psi }_{b})$$

11b $${j}_{y}=2{v}_{F}{\rm{Re}}({\psi }_{a}^{\ast }{\psi }_{b})$$

with

12 $${\psi }_{a}^{\ast }{\psi }_{b}={|A|}^{2}{z}_{+}{e}^{i({k}_{x}-{k}_{x}^{\ast })}x-{A}^{\ast }B{z}_{-}{e}^{-i({k}_{x}+{k}_{x}^{\ast })x}-{B}^{\ast }A{z}_{+}{e}^{i({k}_{x}+{k}_{x}^{\ast })x}-{|B|}^{2}{z}_{-}{e}^{i({k}^{\ast }x-{k}_{x})x}$$

where *z*_±_ have the same definitions as for the transmission problem and

$${k}_{x}=\frac{1}{{v}_{F}\hslash }\sqrt{{E}^{2}-{J}_{S}^{2}-{({v}_{F}\hslash {k}_{y,n})}^{2}}\in {\mathbb{C}}$$

If no reflection occurs, then *B* = 0 and ${k}_{x}\in {\mathbb{R}}$. Consequently, ${j}_{x} \sim {k}_{x}$ and ${j}_{y} \sim {k}_{y,n}$, both being independent of *x*. As long as *n* ≠ 0 ⇒ *j*_*y*_ ≠ 0.

When reflections occur, *B* ≠ 0 and *k*_*x*_ is either real or imaginary. For ${k}_{x}\in {\mathbb{R}}$,

13 $${\psi }_{a}^{\ast }{\psi }_{b}={|A|}^{2}{z}_{+}-{A}^{\ast }B{z}_{-}{e}^{-2i{k}_{x}x}-{B}^{\ast }A{z}_{+}{e}^{2i{k}_{x}x}-{|B|}^{2}{z}_{-}$$

We also know that due to current conservation **∇** ⋅ **j** = 0. Consequently, *j*_*x*_ is independent of *x* and *j*_*y*_ will be an oscillatory function of *x*. Therefore, finite *j*_*y*_ can be obtained, even if *n* = 0. This is due to the spin-momentum locking mechanism of the TI. When *k*_*x*_ is imaginary, *j*_*y*_ will be an exponentially decaying function of *x*. For a general **m**(*x*,*y*) the above formalism is not valid and both probability current density components are a function of *x* and *y*, always satisfying the continuity equation.

### Transmission problem-Numerical

The time-independent Schrödinger equation, *H*Ψ = *E*Ψ, is discretized using finite difference method on a rectangular grid with *N*_*x*_ and *N*_*y*_ segments along $\hat{{\bf{x}}}$ and $\hat{{\bf{y}}}$ accordingly. For the trivial magnetization texture, *N*_*x*_ and *N*_*y*_ can be smaller than that for a non-trivial texture, something which translates into shorter computation times. Specifically, for the trivial texture we have used *N*_*x*_ = *N*_*y*_ = 80 while for a single skyrmion texture, *N*_*x*_ = *N*_*y*_ = 120. In general the more **m**(*x*,*y*) varies as a function of *x* and *y*, the higher degree of discretization is required in order to resolve this rapid change

The wavefunction is a spinor, $${\rm{\Psi }}(x,y)={(\begin{array}{cc}{\psi }_{a}(x,y) & {\psi }_{b}(x,y)\end{array})}^{T}$$. For the effective hamiltonian which we are using, vanishing boundary conditions are not possible due to the nature of the Dirac Eq. (1). To emulate the conducting side surfaces of the 3D TI, we impose periodic boundary conditions on our wavefunction. This results into the transverse eigenmode taking on discrete values: *k*_*y*,*n*_ = 2*nπ*/*W* with $n\in {\mathbb{Z}}$. For the width values we are considering, only few transverse modes are available to support travelling waves along the $\hat{{\bf{x}}}$ direction.

The spinor components can be expanded in terms of the transverse eigen-modes. At the input, $x=-\tfrac{L}{2}$, $y\in [-\tfrac{W}{2},\,\tfrac{W}{2}]$, the wavefunction will read

14a $${\psi }_{a}^{(in)}(\frac{-L}{2},y)={e}^{-iL\frac{{k}_{x,n}}{2}}{\varphi }_{n}(y)+\sum _{m\in {\mathbb{Z}}}{r}_{m}{e}^{iL\frac{{k}_{x,m}}{2}}{\varphi }_{m}(y)$$

14b $${\psi }_{b}^{(in)}(\frac{-L}{2},y)={e}^{-iL\frac{{k}_{x,n}}{2}}{\varphi }_{n}(y){z}_{1}(n)-\sum _{m\in {\mathbb{Z}}}{r}_{m}{e}^{iL\frac{{k}_{x,m}}{2}}{\varphi }_{m}(y){z}_{2}(m)$$

where ${r}_{m}\in {\mathbb{C}}$ is the reflection amplitude, ${\varphi }_{m}(y)=\sqrt{1/W}{e}^{i\tfrac{2m\pi }{W}y}$ is the transverse eigen-mode with the index *n* refering to the input eigen-mode and

$${z}_{\mathrm{1,2}}(m)=\frac{{v}_{F}\hslash }{E}(i{k}_{x,m}\mp {k}_{x,m})$$

$${k}_{x,m}=\{\begin{array}{ll}\frac{1}{{v}_{F}\hslash }\sqrt{{E}^{2}-{({v}_{F}\hslash {k}_{y,m})}^{2},} & |m|\le {{\mathcal{N}}}_{max}\\ i\frac{{v}_{F}\hslash }{E}\sqrt{{({v}_{F}\hslash {k}_{y,m})}^{2}-{E}^{2},} & |m|\ge {{\mathcal{N}}}_{max}+1\end{array}$$

with ${{\mathcal{N}}}_{max}$ is the maximum number of transverse modes that have been included in the simulations. For the present results, ${{\mathcal{N}}}_{max}=5$. The scattering that occurs due to the exchange interaction is elastic. Consequently, at the other interface, *x* = *L*/2 the wavefunction is a linear combination of waves with energy *E*.

15a $${\psi }_{a}^{(out)}(\frac{L}{2},y)=\sum _{s\in {\mathbb{Z}}}{t}_{s}{\varphi }_{s}(y){e}^{iL\frac{{k}_{x,s}}{2}}$$

15b $${\psi }_{b}^{(out)}(\frac{L}{2},y)=\sum _{s\in {\mathbb{Z}}}{t}_{s}{\varphi }_{s}(y){z}_{1}(s){e}^{iL\frac{{k}_{x,s}}{2}}$$

where ${t}_{s}\in {\mathbb{C}}$ is the transmission amplitude. In order to incorporate the boundary conditions, we employ the Quantum Transmitting Boundary Method^56^ which we modified for the Dirac equation. Exploiting the orthogonality of the transverse eigenmodes, we express the reflection and transmission amplitudes as follows:

16a $${r}_{m}=\langle {\varphi }_{m}(y)|{\psi }_{a}(\frac{-L}{2},y)\rangle {e}^{-iL\frac{{k}_{x,m}}{2}}-{\delta }_{m,n}{e}^{-i{k}_{x,n}}={I}_{\mathrm{1,}m}{e}^{-iL\frac{{k}_{x,m}}{2}}-{\delta }_{m,n}{e}^{-iL{k}_{x,n}}$$

16b $${t}_{s}=\langle {\varphi }_{s}(y)|{\psi }_{b}(\frac{L}{2},y)\rangle {e}^{-iL\frac{{k}_{x,s}}{2}}\frac{1}{{z}_{1}(s)}=-{I}_{\mathrm{2,}s}{e}^{-iL\frac{{k}_{x,s}}{2}}{z}_{2}(s)$$

The QTBM conditions will be given by inserting Eq. (16a) into Eq. (14b) and Eq. (16b) into Eq. (15a):

17a $${\psi }_{b}^{(in)}(-\frac{L}{2},y)=2\,i\,{k}_{\chi ,n}{e}^{-iL\frac{{k}_{x,n}}{2}}{\varphi }_{n}(y)-\sum _{m\in {\mathbb{Z}}}{I}_{\mathrm{1,}m}{\varphi }_{m}(y){z}_{2}(m)$$

17b $${\psi }_{a}^{(out)}(\frac{L}{2},y)=-\sum _{s\in {\mathbb{Z}}}{I}_{\mathrm{2,}s}{\varphi }_{s}(y){z}_{2}(s)$$

The probability current density *j*_*x*_(*x*) = *qvj* ∫d*y* $\langle {\rm{\psi }}|{\sigma }_{{y}}|{\rm{\psi }}\rangle$ and the transmission probability is then

$${T}_{n}(E)\equiv \frac{{j}_{x}^{(out)}}{{j}_{x}^{(in)}}=\frac{1}{{k}_{\chi ,n}}\sum _{s\in {N}_{t}}{|{t}_{s}|}^{2}{k}_{\chi ,s}$$

where *N*_*t*_ is the set of all input modes *m* such that ${k}_{\chi ,m}=\sqrt{{\varepsilon }^{2}-{k}_{\xi ,m}^{2}}\in {\mathbb{R}}$. Then, the total current can be computed as following:

$${I}_{total}=\frac{q}{h}\sum _{n}{\int }_{-\infty }^{\infty }{\rm{d}}E\,{T}_{n}(E)({f}_{in}(E)-{f}_{out}(E))$$

where *f*_*in*,*out*_(*E*) is the Fermi-Dirac distribution for the input and output contacts. The input modes *n* over which we do the summation are all transverse eigen-modes which satisfy the following condition:

$${k}_{x,n}=\frac{1}{{v}_{F}\hslash }\sqrt{{E}^{2}-{({v}_{F}\hslash {k}_{y,n})}^{2}} > \,0$$

Equivalently, the sum is done over all the modes below the line *E* = *E*_*f*,1_ in Fig. 2 for which ${k}_{x}\in {\mathbb{R}}$ can be defined, because for *E* < *v*_*F*_ħ*k*_*y*,*n*_, ${k}_{x}\in {\mathbb{C}}$ and the input wave decays exponentially as a function of *x*.

Finally, the conductance can be computed using *G* = d*I*_*total*_/d*V* = *q*d*I*_*total*_/d*E*_*f*,1_. The reference conductance *G*_0_ for one mode, is defined for ballistic transport, i.e. *T*(*E*) = 1 in the low-temperature limit: *G*_0_ = *q* ^2^/*h*. Moreover, in the low-temperature limit, we obtain *G* = *G*_0_∑_*n*_ *T*_*n*_(*E*). Consequently, for one input transverse mode, the transmission probability is equivalent to the conductance ratio. For the trivial magnetization texture as well as for magnetization textures where analytical solutions are possible, the numerical procedure yields the same results. This ensures the validity of the the QTBM conditions.

### Pearson correlation coefficient

The Pearson correlation coefficient (also known as Pearson’s *r*, or Pearson product-moment correleation coefficient), is a statistical measure of the linearity between two datasets *X* and *Y*. Its formal definition is the ratio of the covariance between the two variables and the product of their standard deviations:

$$r=\frac{{\rm{cov}}(X,Y)}{{\sigma }_{X}{\sigma }_{Y}}$$

A value of the coefficient close to 1 (−1) implies a linear (anti-linear) behavior between the variables *X* and *Y*. On the other hand, a value of the coefficient close to 0 means that there is no linear relation between the variables. For our current study, the datasets *X* and *Y* at each input voltage point are comprised of four elements. More specifically,

$$X\equiv \eta =\{\pi \mathrm{/4,}\pi \mathrm{/8,}\pi \mathrm{/9,}\pi \mathrm{/18\}}$$

and

$$Y=\{{\rm{\Delta }}G{(V)}^{(\eta =\pi \mathrm{/4)}},{\rm{\Delta }}G{(V)}^{(\eta =\pi \mathrm{/8)}},{\rm{\Delta }}G{(V)}^{(\eta =\pi \mathrm{/9)}},{\rm{\Delta }}G{(V)}^{(\eta =\pi \mathrm{/18)}}\}$$
